# Impact of Application of Selected Composite Materials on the Weight and Vibroactivity of the Upper Gearbox Housing

**DOI:** 10.3390/ma12162517

**Published:** 2019-08-07

**Authors:** Tomasz Figlus, Mateusz Kozioł, Łukasz Kuczyński

**Affiliations:** 1 Faculty of Transport, Silesian University of Technology, 40-019 Katowice, Poland; 2 Faculty of Materials Engineering and Metallurgy, Silesian University of Technology, 40-019 Katowice, Poland

**Keywords:** gearbox, fiber-reinforced polymer composite, vibration, noise

## Abstract

In this paper, exploratory studies on the application of fibre-reinforced polymer composites in the construction of gearbox housing elements that are used for transport means, as an alternative to traditionally used materials, were undertaken. Composite materials with three types of reinforcement were used: a glass-chopped strand mat, glass fabric, and carbon fabric. The manufactured elements were subjected to weight assessment and vibroactivity tests, including the recording of vibration and noise. The obtained results were compared to the values recorded for housings made of steel. It has been found that composite housings, while maintaining geometric similarity, are characterised by at least 60% lower weight compared to steel housings. It has been shown that in the frequency range below 1 kHz, composite housings are characterised by the presence of resonant frequencies with higher amplitudes than steel housings. At higher frequency ranges—above 1 kHz—composite housings had a lower vibroactivity level than steel housing. They allowed a significant reduction in the level of vibration and noise in this frequency range. The results obtained indicate that composite gearbox housings can be a good alternative to steel-based solutions.

## 1. Introduction

Vibration and noise are among the main environmental impacts of means of transport [1,2,3,4]. Their sources are, among others, the interaction of individual elements, aerodynamics, and friction. The research and analysis of these interactions make it possible to indicate whether and to what extent the generated vibroacoustic signals create a nuisance or are harmful [1,2,3,5,6,7,8,9,10,11].

Gearboxes belong to the basic components of the powertrain system. The interaction of their individual elements results in vibration and noise [5,12,13,14,15,16,17,18], which on the one hand are undesirable effects of their operation, and on the other hand contain important information, e.g., diagnostic information about the technical condition [19,20,21,22,23,24,25,26,27,28,29,30]. The use of gearboxes in drive trains leads to a significant increase in their weight. This phenomenon is undesirable, particularly in view of the energy consumption of means of transport.

Studies conducted on the vibroactivity of gearboxes so far have focused on the possibility of reducing vibration and noise levels [31,32,33,34], while allowing a small increase in weight [35,36]. It has been proven that the selection of specific geometrical features of the gearing [33,37,38,39] and the additional ribbing of the housing reduces the vibroactivity of the gearbox [34,36,40,41,42,43,44], but with a slightly increase in weight [36,45]. However, such an approach in the construction of e.g., motor vehicles is not advisable, as it directly contributes to increases in fuel consumption. 

The possibility of using new types of materials for the construction of the elements of the means of transport in many cases makes it possible to increase their durability and reliability, and contributes to a decrease in weight. One of the types of materials that are characterised by very good mechanical properties at relatively low weight is fibre-reinforced polymer (FRP) composites. It has been observed that the use of this material type in the construction of means of transport plays an increasingly important role [46,47]. The interest of the industry in the possibilities of reducing the weight of means of transport while maintaining a similar level of vibroactivity and strength of the structure is becoming very important [46,48]. Therefore, composite materials can be a good alternative to the basic materials that are used in the construction of means of transport. 

The studies conducted on composite materials so far have focused mainly on manufacturing technologies and strength tests [49,50,51,52,53]. According to the information available in the scientific and industrial literature, composite materials have not yet been subjected to detailed vibroacoustic tests. Some works in this area have been published by the authors of this study, among others [54,55]. In the literature, there is a distinct lack of manufacturing and functional guidelines concerning the vibroacoustic properties of composite materials that can be used in the construction of means of transport and gearboxes.

In this paper, exploratory studies on the application of composite materials in the construction of the element of the gearbox housing designed for means of transport, as an alternative to currently used materials such as steel, were undertaken. It has been assumed that, as part of the research, housings made of three representative types of FRP composite materials would be tested and analysed. The paper analyses the influence of these materials on the vibroactivity and weight of the housings while maintaining the same geometric dimensions. The results obtained are to serve as a source of basic information on the vibroactivity and change in weight of gearbox housings made of composite materials of a given type.

## 2. Materials and Methods

### 2.1. Stand for Testing Gearbox Housings

**Figure 1:**
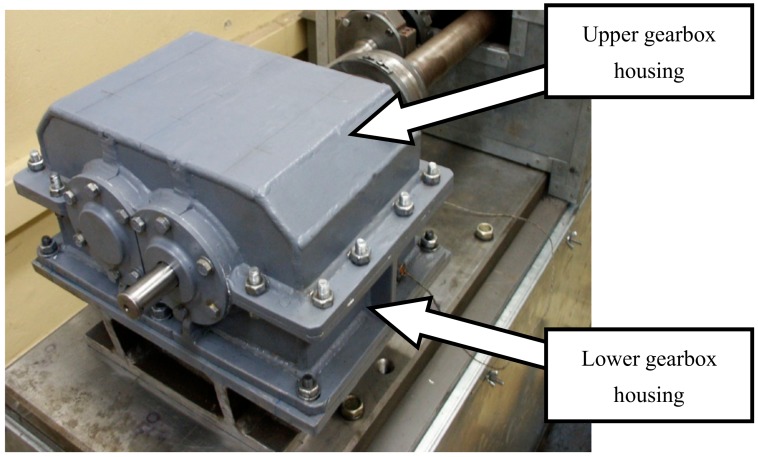
View of the gearbox tested on the FZG power circulating stand.

An FZG power circulating test stand (Power Transmission Systems Laboratories at the Faculty of Transport, the Silesian University of Technology, Katowice, Poland,) was selected for testing gearbox housings made of various materials. This stand (Figure 1) is equipped with a gear and allows experiments to be carried out, among others, with the use of various gearbox housing design solutions. Examples of results of research conducted on the stand were published in papers [35,37,45]. The upper part of the gearbox housing is an interchangeable element in this study. The subject of the study is the assessment of the influence of various materials used in the housing on its vibroactivity and weight. 

### 2.2. Assumptions for Composite Gearbox Housings

Research into the possibility of using different composite materials for gearbox housings required the adoption of preliminary assumptions in which the following issues were considered:

- Technological possibilities of manufacturing the housings;
- Economic viability of using composite materials to manufacture the housings;
- Structural safety when using the proposed materials;
- Possibility of carrying out tests on the manufactured housings.

The basic assumption is that the geometric shape of housings made of new materials is to be as unchanged from a steel housing as possible, and that the housings will not be damaged or destroyed during tests. On this basis, the following assumptions for the manufacture of composite gearbox housings were developed:

- The shape of the housings will correspond to that of the steel housing in accordance with geometric similarity assumptions;
- Modifications may be introduced only when this is required in connection with the technological considerations of the composite manufacturing process;
- The housings are to be manufactured from three different representative composite materials with known physical and mechanical properties;
- The material of the composite housings is not to be affected by temperature during experiments;
- The weight of composite housings is to be significantly lower than that of the steel housing;
- Bearing nodes will be made of steel and will be glued into the housings.

### 2.3. Manufacture of Composite Gearbox Housings

After adopting the assumptions presented in this section, the production of three different housings was started. These housings were manufactured at the Faculty of Materials Engineering and Metallurgy of the Silesian University of Technology (Katowice, Poland) and at the Silesian Science and Technology Centre of Aviation Industry in Czechowice-Dziedzice (Poland). 

Two composite materials reinforced with glass fibre, with different forms of reinforcement: fabric and a chopped strand mat were selected for the tests. These types of reinforcement represent two extreme reinforcing models for a shell-shape element that is isotropic and orthotropic. Assuming a constant geometry of the product, this resulted in a difference in basis weight and the number of layers. ESTROMAL 14 LM polyester resin with METOX 50 catalyst (LERG, Pustkow, Poland) was used as the matrix for these composites. The bodies were made manually with a vacuum bag; the description of their manufacture is included in the paper [56]. The third composite was reinforced with an 800 g/m^2^ carbon fabric with an epoxy resin matrix. The product was formed using an autoclave technique from DELTA PREG’s (Italy) prepregs. The material description of the manufactured housings is provided in Table 1. Carbon fiber composite laminates formed in an autoclave can be applied for responsible elements. The form of fabric enables some comparison with the glass fiber composite. 

Manufactured composite housings without bearing nodes were characterised by significantly lower weight compared to a steel housing (with bearing nodes), respectively: Steel housing/K_1 housing → 13 kg/1.3 kg, Steel housing/K_2 housing → 13 kg/2.1 kg, Steel housing/K_3 housing → 13 kg/1.6 kg. 

**Figure 2:**
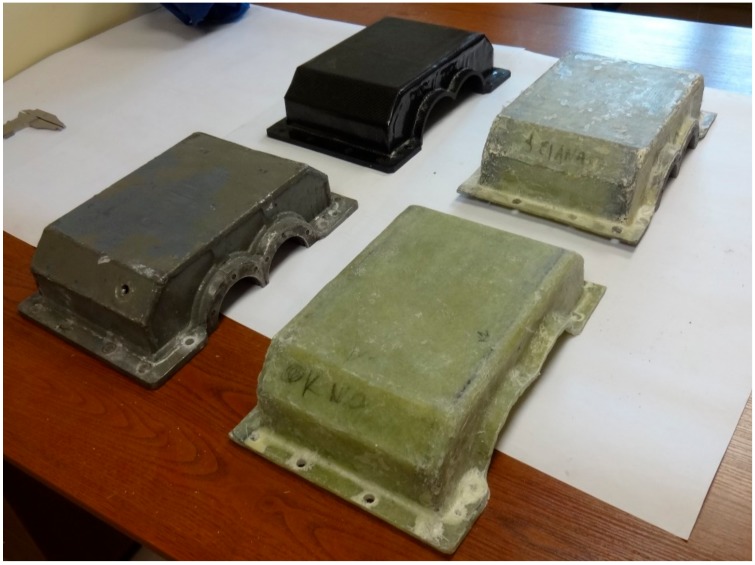
Steel housing (with bearing nodes) and composite housings before mounting bearing nodes.

Figure 2 shows the view of the housings after the manufacturing process.

**Figure 3:**
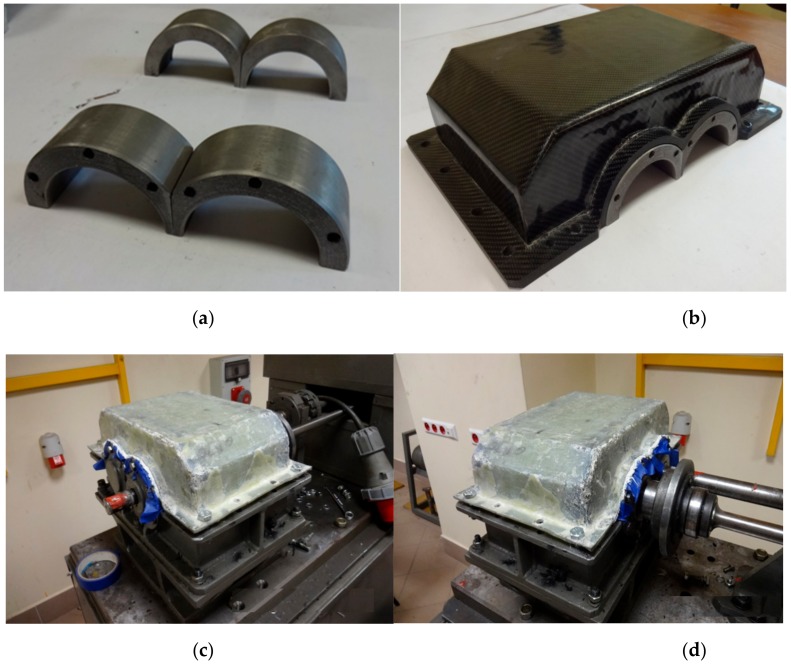
Bearing nodes: (**a**) before mounting, (**b**) K_3 housing with bearing nodes prepared for gluing, (**c**) and (**d**) fitting and stiffening of the K_1 housing during the process of gluing bearing nodes.

In order to make it possible to fit the manufactured composite housings on the stand, bearing nodes made of steel St3 were glued into them. Bearing nodes were not made of composites at the presented stage of the tests due to the need to ensure the safety of the structure; this is mainly about the transfer of dynamic stresses from bearings and shafts. Attempts to make such elements out of composites are scheduled for further stages of the research. The view of the bearing nodes before mounting is shown in Figure 3a,b. In order to make sure that the housings were properly pressed against bearing nodes and ensure the parallelism of axes, the gluing process was performed directly at the stand, an example of which is shown for K_1 housing in Figure 3c,d.

After gluing the bearing nodes into the composite housings, their weight increased respectively to a value of: K_1 = 4.4 kg, K_2 = 5.2 kg, and K_3 = 4.7 kg (Table 2). However, the weight of composite housings is still at least 60% lower than that of the steel housing. 

### 2.4. Modal Analysis of Housings Mounted on the Stand

An experimental modal analysis was used to evaluate the dynamic properties of the manufactured gearbox housings. The response of the motion of the system to impulse induction will be searched for. The transition function (TFE) [57] was determined on the basis of that response. This function is estimated using the Welsh algorithm, which can be used to identify non-parametric systems. This method estimates the information contained both in the amplitude and phase of signals. 

The function of the transition between two signals x(n) and y(n) is determined on the basis of the dependence (1):

(1) $$\hat{H}\left(\omega \right)=\frac{{\hat{P}}_{xy}\left(\omega \right)}{{\hat{P}}_{xx}\left(\omega \right)}$$

where:

${\hat{P}}_{xy}\left(\omega \right)$—common power spectral density (CSD) of signals x(n) and y(n),
${\hat{P}}_{xx}\left(\omega \right)$—power spectral density (PSD) of signal x(n).

**Figure 4:**
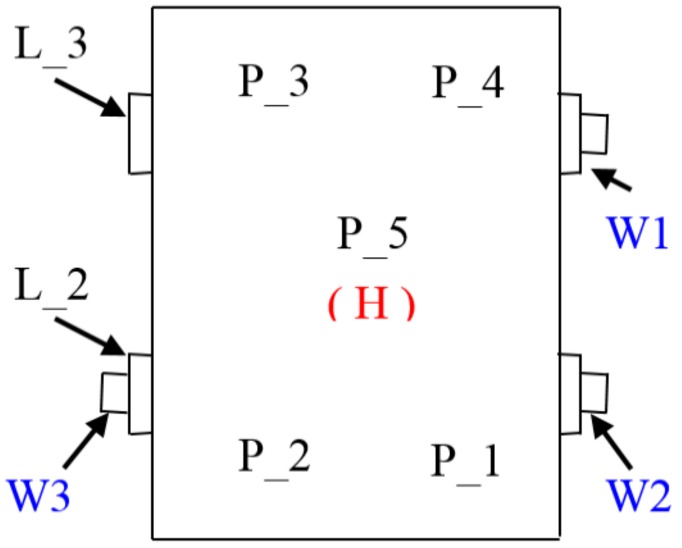
Arrangement of the points of forces impact (L)–(W)–(P) and measurement of vibration (P) and noise (H).

Figure 4 shows the arrangement of the points of induction and measurement of vibration and noise. 

The examination of the resonance structure of the gearbox housing mounted on the measuring stand makes it possible to determine the modal frequencies of vibration and noise present during normal operation (Figure 4). The modal frequency differences between a stand-mounted housing and a free-hanging housing are caused by the stiffening of the structure after mounting it on the stand and loading it with wheels, bearings, and shafts, as well as by the presence of oil. 

**Figure 5:**
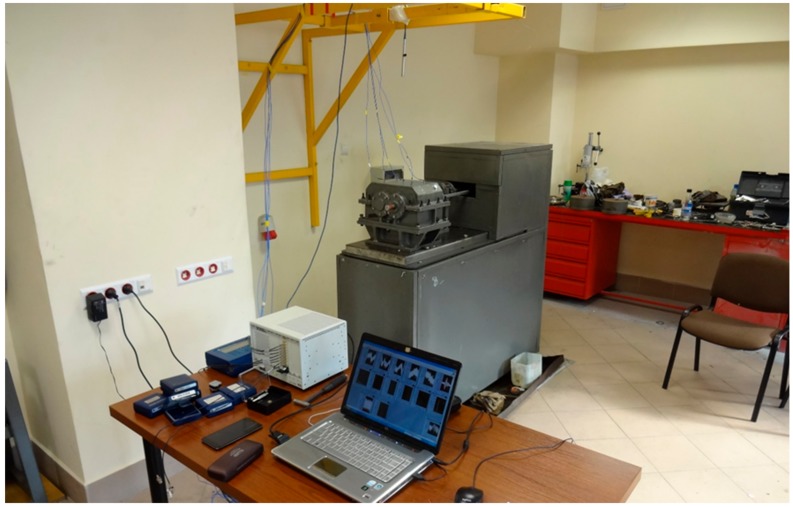
View of the FZG stand and the measuring system for stand tests.

**Figure 6:**
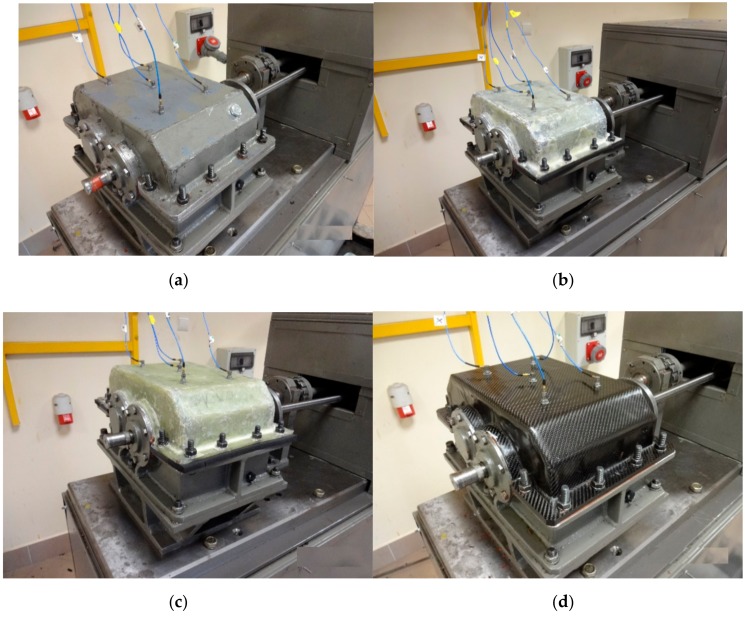
View of housings mounted on the stand together with measuring transducers: (**a**) Steel housing, (**b**) K_1 housing, (**c**) K_2 housing, and (**d**) K_3 housing.

Vibroactivity tests were carried out on the measuring stand shown in Figure 5 and Figure 6. In the experiment, impulse induction was applied with a pulse hammer equipped with a force transducer, five piezoelectric transducers to record the normal vibration acceleration of the upper plate of the housing, and a directional microphone positioned 0.5 m from the upper cover of the housing. Impulse induction with a modal hammer was applied in the immediate vicinity of P_1–P_5 measuring transducers. During the working measurement, induction was applied via shafts W2 and W3 (Figure 4). This made it possible to obtain characteristics of natural and mutual vibration, as well as the function of the transition between the vibration excitation points and the measurement points located on the upper plate of the housing. 

During the experiments, all the signals were recorded simultaneously at a sampling rate of 20 kHz. The measurements used the National Instrument NI 4472 data acquisition card, LabView software (Academic Site License Research Standard Service Program, National Instruments Poland, Warszawa, Poland), and Matlab software (Academic Individual Perpetual, Oprogramowanie Naukowo - Techniczne sp. z o.o., Kraków, Poland) for signal processing. 

## 3. Results and Discussion

**Figure 7:**
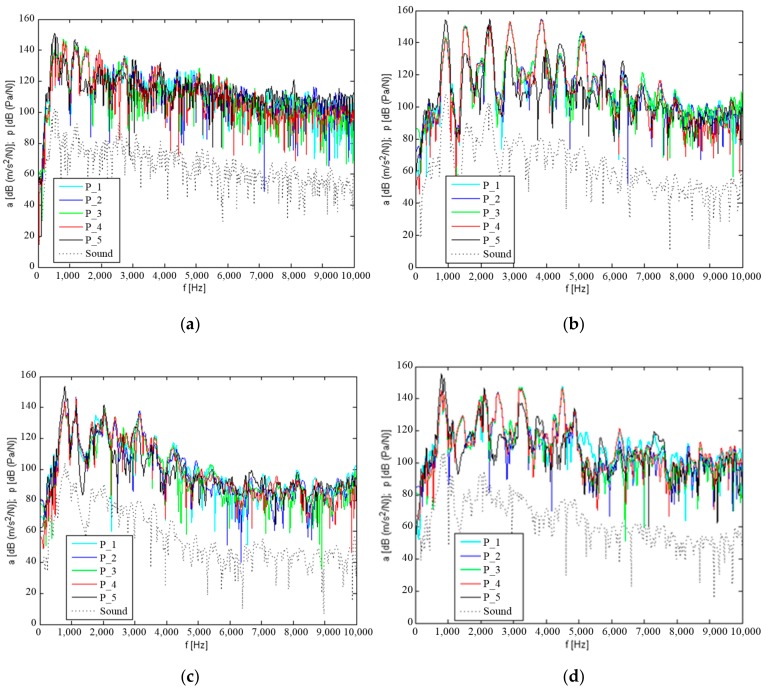
Natural and mutual transmittance of vibration response to impulse induction at point P_1 and sound level above the housing: (**a**) Steel housing, (**b**) K_1 housing, (**c**) K_2 housing, and (**d**) K_3 housing.

**Figure 8:**
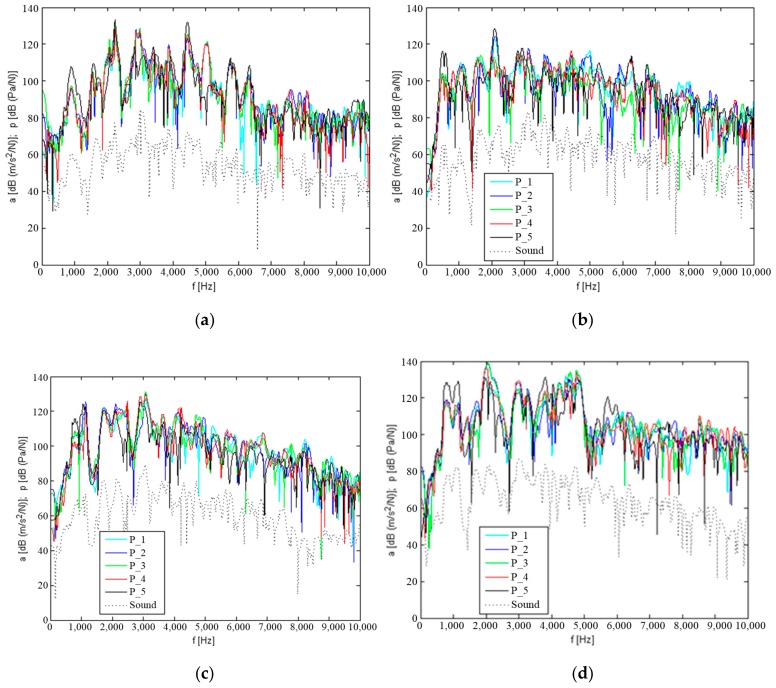
Natural and mutual transmittances of vibration response to impulse induction at point W2 and sound level above the housing: (**a**) Steel housing, (**b**) K_1 housing, (**c**) K_2 housing, and (**d**) K_3 housing.

Figure 7 and Figure 8 show the determined natural and mutual transmittances of the vibration response of the measurement points P_1–P_5, which are located on the upper plate of the tested housings, with impulse excitation applied at point P_1 and on shaft W2. 

The results obtained indicate that the steel housing mounted on the stand was characterised by the highest number of resonant frequencies occurring in the frequency range up to approximately 5.5 kHz, with a similar amplitude. Only above this frequency is a significant damping of vibration observed on the transmittance characteristics. With K_1 and K_2 composite housings, the highest amplitude resonances occur at frequencies below 1 kHz, and the resonance amplitude decreases with increasing frequency. The K_3 composite housing is characterised by resonance with the highest amplitude resonance at frequencies below 1 kHz. However, numerous local amplitudes with significant energy can also be observed in frequency distributions at higher frequencies up to approximately 5 kHz.

Based on the tests performed for vibration induction generated on the W2 wheel shaft, it may be concluded that the damping of vibration from the shaft through the bearings and housing significantly contributes to the lower amplitude level of the determined resonant frequencies. The determined mutual transmittances showed similar resonance frequencies as in the induction generated at point P_1, but with significantly lower amplitude. It can also be observed that many resonant frequencies have clearly different transmittance in terms of the amplitude value. This is due to the damping of vibration on their transmittance route from the shaft to the housing. 

**Figure 9:**
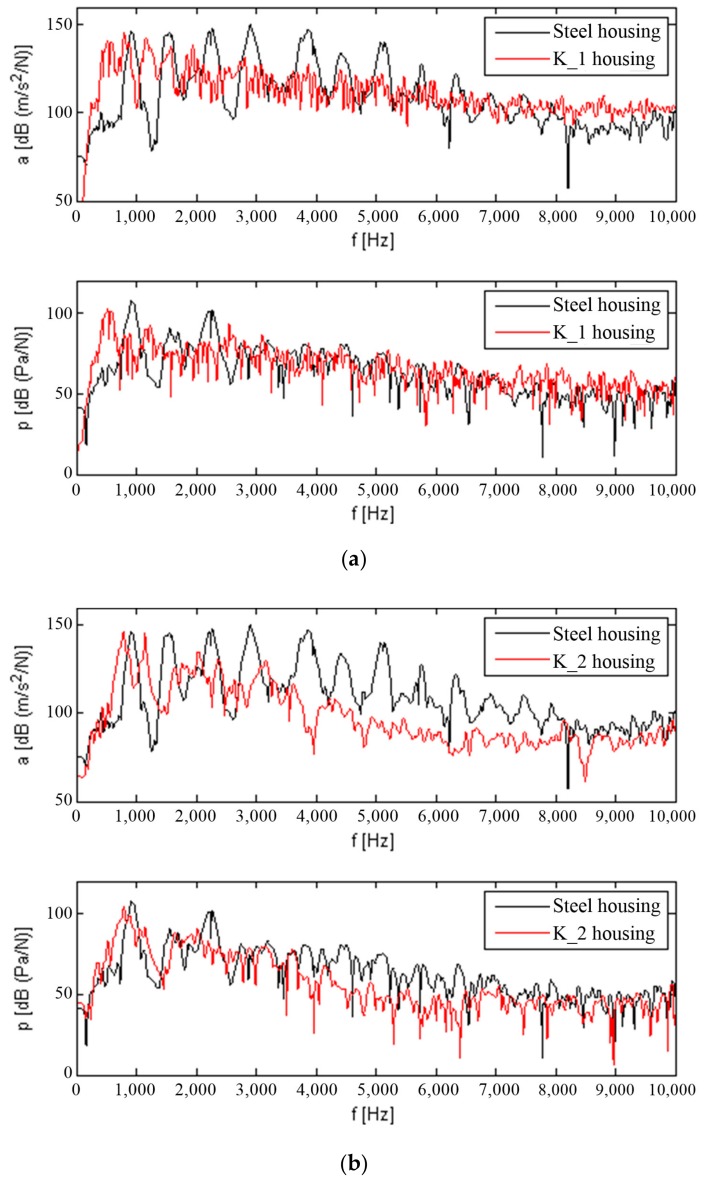

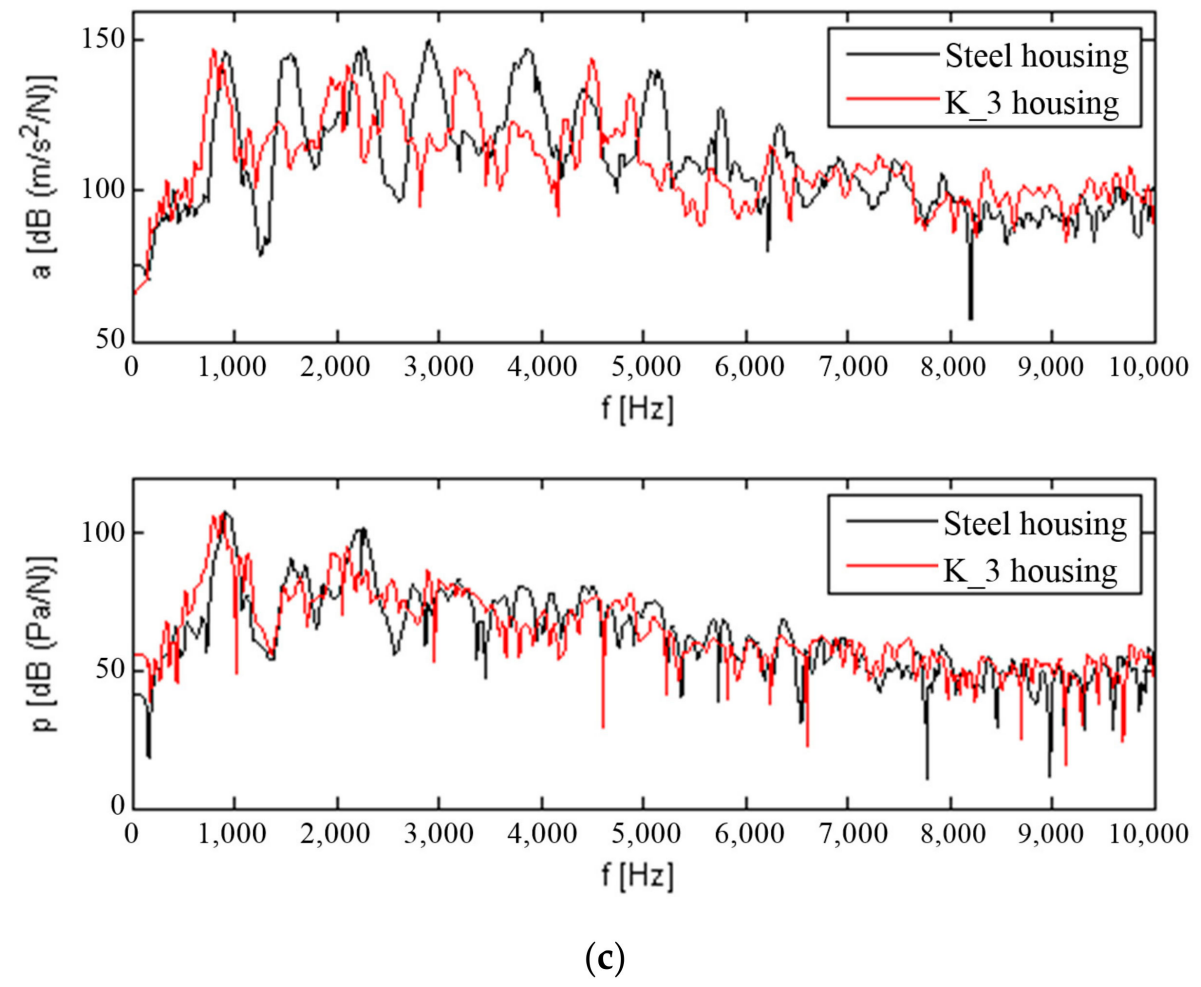
Comparison of averaged vibration acceleration (a) and sound pressure (p) of composite housings and steel housing: (**a**) Steel housing-K_1 housing, (**b**) Steel housing-K_2 housing, and (**c**) Steel housing-K_3 housing.

In order to make qualitative comparisons of the resonance structure of composite housings K_1–K_3 and the steel housing, vibration acceleration values were obtained for five measurement points (shown in Figure 7) and averaged. Figure 9 shows the average levels of the vibration response of the upper plate of the housings and of the sound pressure level from the composite materials compared to the steel housing. 

When analysing the qualitative changes in the transmittance of vibration and noise, it can be observed that after mounting the K_1 and K_2 housings on the stand, the amplitude of their resonant frequencies is significantly lower than those for the steel housing. A significant resonant excitation can only be observed at a lower frequency of up to 1 kHz. The differences between the K_3 housing and the steel housing are smaller.

**Figure 10:**
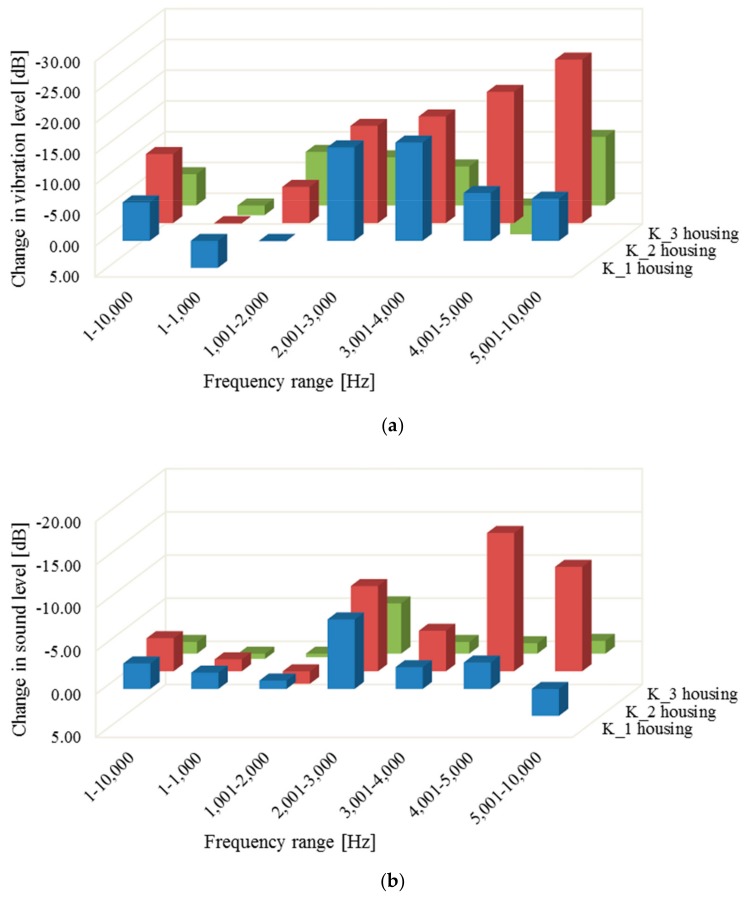
Change in the vibration (**a**) and sound (**b**) levels of composite housings K_1–K_3, relative to the steel housing.

On the basis of the obtained results, quantitative calculations of the changes in the vibration and noise of the examined housings were made in the modal analysis, in the entire frequency range of 1 to 10,000 Hz, and in six different frequency ranges from this band. The average values of vibration and sound pressure levels were calculated. The results of these analyses are presented in Table 3 and Table 4, and in Figure 10.

It can be observed that composite housings in the frequency range up to 1 kHz are in all cases louder than the steel housing. However, at higher band ranges, composite housings have a significantly lower noise and vibration response level than the steel housing. This indicates that the use of composite housings makes it possible to reduce weight and at the same time decrease gearbox vibration in the higher frequencies that correspond to the operating conditions of the vast majority of gearboxes [37,45].

Damping in laminates is caused by the viscoelastic behaviour of the polymer matrix [58]. Although the load is transferred mainly by fibres (also in mat-reinforced composites, if the length of elementary fibres exceeds the critical value [59]), the matrix takes part in its transmission by means of tangential stresses [59,60]. This characteristic makes it possible for the matrix to utilise, in particular, mechanical interactions that cause minor displacements. The best example is precisely the effect of vibration damping by laminates [61].

The results obtained (Figure 10) indicate that the best damping compared to the steel housing is shown by the composite reinforced with a glass mat (K_2). This is undoubtedly due to the lower rigidity of the structure of this material type in the plane. Stochastic arrangement of the fibres in the mat, combined with their discontinuous form, results in a greater share of the polymer matrix in the load transfer, and thus in greater possibilities of absorbing and damping elastic vibration [13]. 

Composites reinforced with fabrics (K_2 and K_3) show a significantly higher modulus of elasticity in the directions of fibre arrangement than composites reinforced with mats [60]. This directional rigidity contributes to the better conduction of elastic waves in those directions where conduction and damping are influenced less by the matrix and more by fibres. 

The carbon fibre-reinforced composite (K_3), apart from the oriented internal structure, has at least a four times higher modulus of elasticity than the composite reinforced with glass fabric. This is an additional feature that is conducive to the good conduction of elastic waves and reduction in the influence of the matrix on the mechanical properties of the material. This leads to worse damping, as shown by the results. 

## 4. Conclusions

The paper presents the results of vibroacoustic tests of gearbox housing elements made of three types of polymer composites. The following main conclusions were drawn.

- The combination of composite housings with steel bearing nodes is ideal for short-term loads, e.g., it makes it possible to carry out laboratory tests of new material solutions for gearbox housings. The long-term use and industrial application of this solution requires additional testing (mainly with regard to fatigue crack resistance).
- The application of FRP composites makes it possible to reduce the weight of gearbox housing elements by at least 60% compared to the corresponding steel components.
- Composite gearbox housing elements have resonant vibration and noise frequencies in the lower ranges (less than 1 kHz) than the corresponding steel elements (less than 5.5 kHz).
- In the frequency ranges above 1 kHz, composite elements clearly damp vibration better than corresponding steel components. At frequencies above 4 kHz, a housing element made of a composite reinforced with a glass mat dampens noise by more than 15 dB better and vibration by nearly 27 dB better than the corresponding steel housing element.
- The composite reinforced with a glass mat was the best in terms of vibration and noise damping among the tested materials making up the gearbox housing elements.
- The results obtained indicate without doubt that further research on the use of FRP composites in gearbox housings is advisable.

## Figures and Tables

**Table 1:** Manufactured upper gearbox housings intended for tests.

Photograph of the Housing	Designation	Basic Information
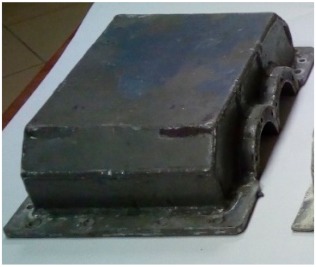	Steel housing	Housing made of steel sheets and sections Material: Steel St3 Manufacture: welded Weight 13 kg (with bearing nodes)
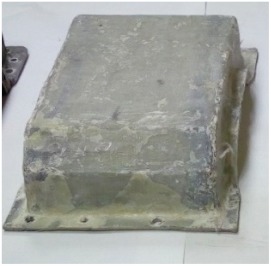	K_1 housing	Housing made of composite reinforced with glass fibre-fabric Material: reinforcement: glass fabric with plain weave, basis weight: 300 g/m^2^, 24 layers; matrix: chemically-hardened polyester resin Manufacture: lay-up with vacuum bag, pressure difference during the process: 0.8 bar, room temperature, curing at room temperature without after-bake Weight 1.3 kg (10% of the weight of the steel housing)
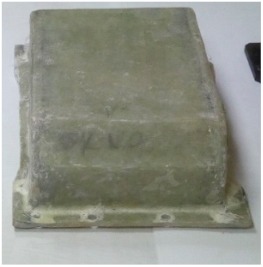	K_2 housing	Housing made of composite reinforced with glass fibre mat Material: reinforcement: glass mat with basis weight of 540 g/m^2^, nine layers; matrix: chemically-hardened polyester resin Manufacture: lay-up with vacuum bag, pressure difference during the process: 0.8 bar, room temperature, curing at room temperature without after bake Weight 2.1 kg (16.1% of the weight of the steel housing)
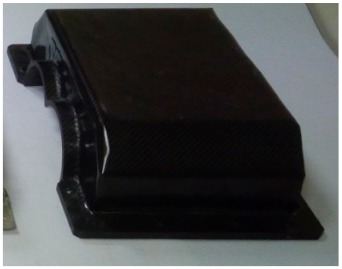	K_3 housing	Housing made of composite reinforced with carbon fibre Material: Prepregs of twill weave carbon fabric (2 × 2) with a basis weight of 800 g/m^2^ (six internal construction layers) and 240 g/m^2^ (external visual layers) and epoxy resin in the precured state Manufacture: Forming in an autoclave, pressure difference during the process: 5 bars, main temperature of the process: 120 °C, process time: 4 h Weight 1.6 kg (12.3% of the weight of the steel housing)

**Table 2:** Weight of upper gearbox housings with glued-in bearing nodes.

Designation	Weight of Housings
Steel Housing	Housing made of steel sheets and sections (with bearing nodes) Weight: 13 kg
K_1 Housing	Housing made of composite reinforced with glass fibre fabric Weight: 1.3 kg + bearing nodes: 3.1 kg = Weight: 4.4 kg 66.2% weight reduction
K_2 Housing	Housing made of composite reinforced with glass fibre mat Weight: 2.1 kg + bearing nodes: 3.1 kg = Weight: 5.2 kg 60% weight reduction
K_3 Housing	Housing made of composite reinforced with carbon fibre Weight: 1.6 kg + bearing nodes: 3.1 kg = Weight: 4.7 kg 63.8% weight reduction

**Table 3:** Change in the vibration level of housings K_1–K_3, relative to the steel housing.

	Change in Vibration Level [dB]		
Frequency Range [Hz]	K_1 Housing	K_2 Housing	K_3 Housing
1 to 10,000	−6.24	−11.25	−5.09
1 to 1000	4.39	0.08	1.58
1001 to 2000	0.06	−5.90	−8.72
2001 to 3000	−15.19	−15.82	−7.84
3001 to 4000	−15.98	−17.35	−6.35
4001 to 5000	−7.78	−21.36	4.68
5001 to 10,000	−6.86	−26.60	−11.16

**Table 4:** Change in the sound level of housings K_1–K_3, relative to the steel housing.

	Change in Sound Level [dB]		
Frequency Range [Hz]	K_1 Housing	K_2 Housing	K_3 Housing
1 to 10,000	−2.96	−3.83	−1.36
1 to 1000	−1.89	−1.39	0.60
1001 to 2000	−0.98	1.45	0.41
2001 to 3000	−8.06	−9.88	−5.83
3001 to 4000	−2.52	−4.70	−1.36
4001 to 5000	−3.06	−16.03	−1.21
5001 to 10,000	3.12	−12.10	−1.48
